# Growth hormone replacement therapy reduces risk of cancer in adult with growth hormone deficiency: A meta-analysis

**DOI:** 10.18632/oncotarget.13251

**Published:** 2016-11-09

**Authors:** Zhanzhan Li, Qin Zhou, Yanyan Li, Jun Fu, Xinqiong Huang, Liangfang Shen

**Affiliations:** ^1^ Department of Oncology, Xiangya Hospital, Central South University, Changsha, Hunan Province 410008, China; ^2^ Xiangya Hospital, Central South University, Changsha, Hunan Province 410008, China

**Keywords:** growth hormone therapy, growth hormone deficiency, cancer, meta-analysis

## Abstract

The risk of growth hormone on cancer in adult with growth hormone deficiency remains unclear. We carried out a meta-analysis to evaluate the risk of cancer in adult with and without growth hormone replacement therapy. We searched PubMed, Web of Science, China National Knowledge Infrastructure, and WanFang databases up to 31 July 2016 for eligible studies. Pooled risk ratio (RR) with 95% confidence interval (CI) was calculated using fixed-or random-effects models if appropriate. The Newcastle-Ottawa Scale was used to assess the study quality. Two retrospective and seven prospective studies with a total of 11191 participants were included in the final analysis. The results from fixed-effects model showed this therapy was associated with the deceased risk of cancer in adult with growth hormone deficiency (RR=0.69, 95%CI: 0.59-0.82), with low heterogeneity within studies (I^2^=39.0%, P=0.108). We performed sensitivity analyses by sequentially omitting one study each time, and the pooled RRs did not materially change, indicating that our results were statistically stable. Begger's and Egger's tests suggested that there was no publication bias (Z=-0.63, P=0.520; t=0.16, P=0.874). Our study suggests that growth hormone replacement therapy could reduce risk of cancer in adult with growth hormone deficiency.

## INTRODUCTION

Growth hormone secreted by the pituitary gland plays important roles in the process of promoting the growth and development for children and regulating the materials metabolism and achieving an energy balance for adult [1]. The adult growth hormone deficiency (AGHD) could cause a series of abnormal manifestations, such as increased body lipid profile, abdominal obesity, impaired glucose tolerance, increased mortality risk of cardiovascular diseases, and so forth [2, 3]. The growth hormone replacement therapy (GHRT) in AGHD patients has been carried out extensively in the last 20 years. Almost all of studies have suggested considerable therapeutic benefits with improvements in lowing the risk of cardiovascular, exercise performance and quality of life [4–6]. Growth hormone has now been approved for children and adult in many countries including Europe, USA and Asia. The safety of growth hormone in the short stage clinical trials is no doubt, but there are still concerns in other clinical setting. The association between GHRT and risk of tumor in adult has remained an important topic.

The hypothesis about the risk of cancer in GHRT patients was based on the biological nature of growth hormone and insulin-like growth factor-1 (IGF-1) [7]. Both experiment and epidemiological have demonstrated that growth hormone and IGF-1 can affect cells in endocrine, paracrine an autocrine manner. It has been proved that the growth promoting function of growth hormone is mainly regulated by IGF-1 [8]. The related signaling transduction cascade induced by IGF-1 receptors can stimulate the cell proliferation and survival [9]. While there are strong evidences from animal and cell experiments suggesting that growth hormone and IGF-1 were involved in the occurrence and development of tumors, such an evidence in human study remains unclear. Several observational studies have suggested an association between IGF-1 level in circulation and a risk of some cancer, such as breast cancer and colorectal neoplasms [10, 11]. Early studies also showed that GHRT was not associated with increased tumor risk. However, a report with 5-year follow-up found that children with GHRT have a higher risk of neoplasms compared with non GHRT. Some studies showed an increased cancer risk [12–14], but others not [15, 16]. The results whether GHRT increased the cancer risk in AGHD patients still remain unclear. The quality and consistency of epidemiological evidence on the topic have not been systematically investigated, which is an important gap in our understanding of the effect of growth hormone on cancer occurrence. Thus, we carry out a meta-analysis to assess the risk of cancer in GHD patients with and without GHRT.

## RESULTS

### Study selection

**Figure 1:**
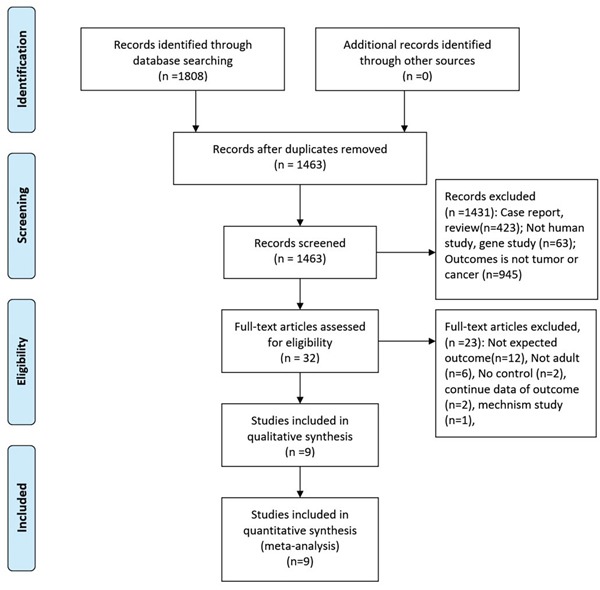
The process of study selection

The initial search returned 1808 records, of which 345 duplicates were excluded. After screening the titles and abstracts, 1431 records were excluded for various reasons (case reports, review, not human study, gene study, unexpected outcomes). Finally, 2retrospective studies and 7 prospective studies were included in the final analysis (see more details in Figure 1) [12–14, 16–21].

### Study characteristics

The main characteristics of included studies are presented in Table 1. Specifically, the studies were published from 2002 to 2013. In total, 11191 participants, 9272 from treatment group, and 1919 from control group were enrolled. The follow-up duration ranged from 2.3 to 14.5 years. Outcomes of 2 studies were craniopharyngioma, and the rest were pituitary adenomas or tumor (see more details in Table 1).

**Table 1:** General characteristics of studies included in the meta-analysis

Author Year	Country	Study design	Age (T/C*)	Male	Dose of GH(UI)	Follow- up(y)	Outcomes	Sample size (T/C)	Outcomes (T/C)
Buchfelder 2007	Germany	Retrospective study	42.1/55.5	-	1.3 UI	5.0	Pituitary adenomas	55/55	18/14
Olsson 2009	Sweden	Prospective study	66.7/66.7	66%	F:0.36 M:0.45mg	10.0	Pituitary adenomas	121/114	31/37
Hatrick 2002	UK	Prospective study	49/52	53.3%	-	3.6	Pituitary tumor	47/28	2/2
Olsson 2012	Sweden	Prospective study	46.6/45.7	53.3%	F:0.72 M:0.45	13.6	Craniopharyngioma	56/70	9/30
Child 2011	USA	Prospective study	46.4/54.4	55.0%	-	3.7	Primary cancers	6840/940	350/71
Arnold 2009	UK	Prospective study	53.7/56.2	59.2%	0.1-0.8mg	6.8	Pituitary adenomas	23/107	8/38
Karativetak 2006	UK	Prospective study	17.6/38.8	61.2%	0.3-2UI	10.8	craniopharyngioma	32/53	4/22
Mackenzie 2011	UK	Retrospective study	33/29	51.4%	-	14.5	Pituitary tumor	110/110	11/11
Hartman 2013	USA	Prospective study	46.0/55.0	59.0%	6-12 ug/kg/d	2.3	Pituitary tumor	1988/442	32/12

* T=treatment, C=Control

### Assessment of quality

The mean score of included studies is 7.1 (6-8), which could be considered to be priority according to the assessment criteria. The overview of the quality of included studies are comparable. The primary flaw of all studies is the lack of data after expected outcomes occur. One study did not give details about baseline characteristic. The Supplementary Table S1 shows these details.

### Pooled results

**Figure 2:**
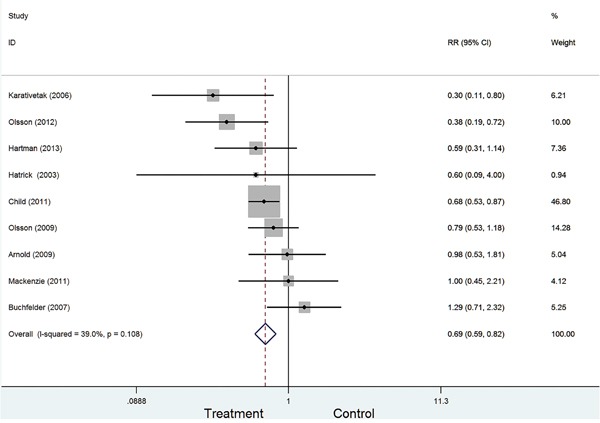
Forest plot for GH replacement therapy for cancer risk in adult with GHD

Figure 2 presented the combined the results from the fixed-effects model. In totally, 11191 study subjects were included in the analysis. The results suggested that GH replacement therapy was associated with the deceased risk of cancer in adult with GHD (RR=0.69, 95%CI: 0.59-0.82), with low heterogeneity across studies (I^2^=39.0%, P=0.108).

**Figure 3:**
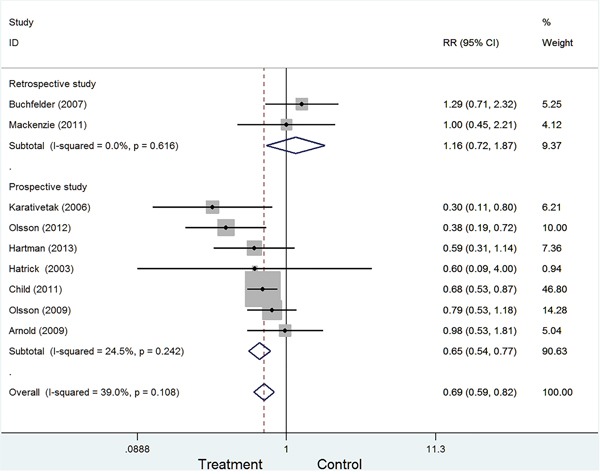
Forest plot of subgroup according to study designed type

To examine the stability of pooled results, we also conducted subgroup analyses by excluding some studies. The following four types were conducted: Type I: excluding 2 retrospective studies (RR=0.65, 95%CI:0.54-0.77, Figure 3); Type II: excluding 2 studies with less than 100 sample (RR=0.72, 95%CI:0.61-0.85). Type III: excluding 2 studies with craniopharyngioma (RR=0.76, 95%CI:0.64-0.91). Type IV: excluding 3 studies with less than 3-year follow-up (RR=0.73, 95%CI:0.57-0.93). All four types showed growth hormone replacement therapy was associated with a decreased risk of cancer.

### Sensitivity analysis

**Figure 4:**
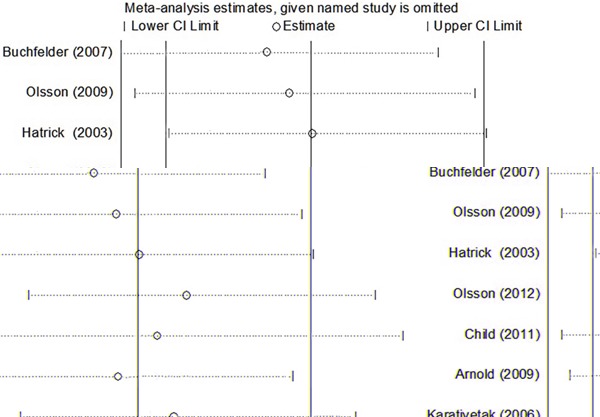
Sensitivity analysis from fixed-effects estimates

We performed a sensitivity analysis by sequentially omitting one study each time, and the pooled RRs did not materially change, indicating that our results were statistically stable (Figure 4).

### Publication bias

**Figure 5:**
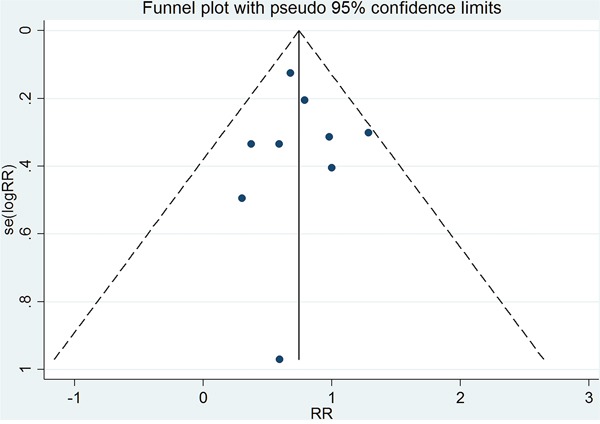
Funnel plot for GH replacement therapy reduces risk of cancer in adult with GHD

As shown in Figure 5, the funnel plot is relatively symmetrical. Begger's and Egger's tests suggested that there was no publication bias (Z=-0.63, P=0.520; t=0.16, P=0.874).

## DISCUSSION

Our results suggest that growth hormone replacement therapy reduces risk of cancer in adult. In addition, the association was also consistent in sensitivity analyses.

The association between GH-IGF-1 and tumor shows a huge difference among vitro, animal experiments and epidemiological investigation. In vitro, growth hormone can stimulate lymphocytes to lymphoblast, growth hormone and its receptors were expressed in almost cancer cells. Over-expression of growth hormone could promote cell proliferation and apoptosis reduction for breast cancer. The IGF-1 also has proliferation and anti-apoptosis property for all types of cell. IGF-1 induces human leukemia cell proliferation and increased DNA replication of liver cell tumor in rat. This function can be inhibited using related antagonist inhibits [22]. Besides, IGF-1 in circulation can be combined with all kinds of binding protein, such as insulin-like growth factor binding protein 3 [23]. With different from IGF-1, insulin-like growth factor binding protein 3 can limit the bioactivity of IGF-1, and exerts its action of inhibiting tumor cell growth [24]. In animal experiments, selective knockout of IGF-1 gene causes reduction of IGF-1 level in circulation, and occurrence rate of breast cancer significantly decreased. Also, IGF-1 has a potential of promoting neoplasm metastasis [25]. Many epidemiology studies also mentioned IGF-1 level in plasma is associated with an increased risk of cancer [9, 10]. However, epidemiology studies did not found such an association in human investigation. All of studies included in the meta-analysis reported that growth hormone therapy is not associated with an increased risk of tumor occurrence or recurrence. Child et al found that the overall primary cancer risk in 6840 patients receiving growth hormones adults did not increase, but elevated standardized incidence were found for subgroups in the USA cohort defined by age <35 years [13]. Hartman conducted a prospective study with 1988 growth hormone-treated and 442 untreated GHD patients, and there was no evidence for a growth hormone therapy effect on cancer [17]. Buchfelder also found growth hormone substitution should not be withheld in deficient patients. But a period of 5 years may not have been long enough to verify this influence on recurrence potential [21]. In parallel with these above study, the latter study found unrelated results [12]. On the contrary, our results even found that growth hormone therapy is associated with a decreased risk of the whole group. This finding is the same as the Olsson and his colleagues' report that long-term (10 years) use of growth hormone in hypopituitarism may be considered to be safe in patients with residual pituitary adenomas [19]. Although we do not exactly how this results happen, the present findings hinted that growth hormone therapy are acceptable and safety under the evidence.

Our meta-analysis has some strengths. This study was in accordance with the guidelines of Meta-analysis of Observational Studies in Epidemiology guideline. Also, all included studies in the meta-analysis were cohort studies with a mean score of 7.1, which was quite high-quality. Besides, heterogeneity within studies is quite low, and the combined results could minimize the likelihood of some bias. Both Begger's and Egger's test suggested that there was no publication bias, which further showed the stability of results. Several limitations of this meta-analysis merit consideration. First, the sample size of two studies included studies is less than 100, and may have a low statistical power. Second, the outcome is pituitary tumor and craniopharyngioma, and the present results may be inappropriate for other population setting. Third, two studies treated tumor recurrence as the outcome. Considering the difference between population with secondary and primary tumor, this factor may have some influence on the pooled results. Patients with secondary tumor usually have weak physical condition, and tend to have tumor. After reviewing these studies, both treatment and control group of two studies are those diagnosed with tumor. This balance between two group could reduce some bias. Furthermore, our results were based on unadjusted data, these results could be affected by confounders, and we could not assess the impact of GHRT on other clinical outcome events. Further large-scale controlled surveys are needful.

In conclusion, our results suggest that growth hormone replacement therapy reduces risk of cancer in adult with growth hormone deficiency. Future study with more long-term follow-up are needed to explore the association between GHRT and recurrence of cancer or other types of tumor.

## MATERIALS AND METHODS

### Literature search

We conducted the meta-analysis and systematic review in accordance with the guidelines of Meta-analysis of Observational Studies in Epidemiology guideline (MOOSE, Supplementary Table S2) [26]. We searched PubMed, Web of Science, China National Knowledge Infrastructure (CNKI), and WanFang databased from inception to 31 July 2016 for eligible studies, using the following MeSH Terms: ‘Hypopituitarism’ OR ‘Growth hormone’ OR ‘Growth hormone deficiency’ OR ‘Growth hormone drug effect’ OR ‘Growth hormone therapeutic’ OR ‘GHD’ OR ‘GH deficiency’ and ‘Caner’ OR ‘Tumor’ OR ‘neoplasm’. We also searched the reference lists of previous related reviews for further studies.

### Selection criteria

Two authors (LZZ and LYY) independently carried out the initial search, deleted republication, screened the titles and abstracts for relevance, and identified studies. Any discrepancy was resolved by discussion and consensus.

The included study need meet the following criteria: (1) Study design: we included a retrospective or prospective study (random or semi-random or cohort study) published in English and Chinese; (2) Study population: Adults with GHD according to diagnostic criteria; (3) Intervention: treatment group receives growth hormone replacement therapy, and control had not received growth hormone replacement therapy; (4) Outcome: tumor, cancer or neoplasm during follow-up. When multiple publications were published from the same study, we used the one with the largest sample size.

### Data extraction

We extracted the following data from each study: first author, publication year, country, type of study design, age of study population, male ratio, duration of follow-up, outcomes, sample size, and number of occurrence outcomes. One author (LZZ) extracted data, and another author (LYY) checked them for accuracy.

### Assessment of quality

Two investigators independently used the Newcastle-Ottawa Scale (NOS) to assess the quality of each study [27]. Study with 7-8 adequate items is considered to be priority, equal or more than 5 items is high quality, and study with less than 5 items is considered to be low quality.

### Statistical analysis

The risk ratio (RR) was used as a common measure of the association between GHRT and cancer risk across studies. Heterogeneity was evaluated by using Cochran Q statistic and quantified with I^2^ statistic, which was used to describe the variation of effect size that is attributed to heterogeneity within studies [28, 29]. We considered I^2^ value more than 50% to be significant heterogeneity. Fixed effects models would be selected for I^2^<50% and random-effects would be chosen for I^2^≥50%. We conducted subgroup analyses for exploring the source of heterogeneity, and examining the stability of pooled results. The subgroup analyses were carried out according to four types: Type I: study design (retrospective vs prospective); Type II: sample size (≤100vs. >100); Type III: cancer type (craniopharyngioma vs not); Type IV: duration of follow-up. We used Begg's funnel plots and Egger's regression test to detect publication bias [30, 31]. All statistical analyses were performed by using Stata 14.0 (Corp, College Station TX, USA), P<0.05 was considered to be statistically significant.

## SUPPLEMENTARY MATERIALS TABLES





## Abbreviations

Growth hormone: GH
Adult growth hormone deficiency: AGHD
Growth hormone replacement therapy: GHRT
IGF-1: insulin-like growth factor-1
Risk ration: RR
Confidence interval: CI.
