# Supplementary material for: Growth hormone replacement therapy reduces risk of cancer in adult with growth hormone deficiency: A meta-analysis
Source: Oncotarget. 2016 Nov 9;7(49):81862–9. doi: 10.18632/oncotarget.13251 (PMC5348436; doi:10.18632/oncotarget.13251)
Supplement: Supplementary file 1 [file oncotarget-07-81862-s001.pdf]

Growth hormone replacement therapy reduces risk of cancer in adult with growth hormone deficiency: A meta-analysis

SUPPLEMENTARY TABLES

Supplementary Table S1: Methodological quality assessment (risk of bias) of included studies by Newcastle-Ottawa Scales

Study	Selection				Comparability	Outcome			Total score
	Exposed Cohort	Nonexposed Cohort	Ascertainment of exposure	Outcome of interest		Assessment of outcome	Length of follow-up	Adequacy of follow-up	
Buchfelder 2007	*	*	*	*	*	*	-	-	6
Olsson 2009	*	*	*	*	**	*	-	-	7
Hatrick 2002	*	*	*	-	**	*	-	-	6
Olsson 2012	*	*	*	*	**	*	-	-	7
Child 2011	*	*	*	*	**	*	*	-	8
Arnold 2009	*	*	*	*	*	*	*	-	7
Karativetak 2006	*	*	*	*	**	*	-	-	7
Mackenzie 2011	*	*	*	*	**	*	*	-	8
Hartman 2013	*	*	*	*	**	*	*	-	8

Supplementary Table S2: MOOSE Checklist for Meta-analyses of Observational Studies

See Supplementary File 1
